# Extensive reprogramming of the nascent transcriptome during iPSC to hepatocyte differentiation

**DOI:** 10.1038/s41598-019-39215-0

**Published:** 2019-03-05

**Authors:** Leena E. Viiri, Tommi Rantapero, Mostafa Kiamehr, Anna Alexanova, Mikko Oittinen, Keijo Viiri, Henri Niskanen, Matti Nykter, Minna U. Kaikkonen, Katriina Aalto-Setälä

**Affiliations:** 1 Finnish Cardiovascular Research Center Tampere, Faculty of Medicine and Health Technology, Tampere University, Tampere, 33014 Finland; 2 Prostate Cancer Research Center, Faculty of Medicine and Health Technology, Tampere University, Tampere, 33014 Finland; 3 Tampere Center for Child Health Research, Faculty of Medicine and Health Technology, Tampere University, Tampere, 33014 Finland; 4 A. I. Virtanen Institute for Molecular Sciences, University of Eastern Finland, Kuopio, 70211 Finland; 5 Heart Center, Tampere University Hospital, Tampere, 33520 Finland

## Abstract

Hepatocyte-like cells (HLCs) derived from induced pluripotent stem cells (iPSCs) provide a renewable source of cells for drug discovery, disease modelling and cell-based therapies. Here, by using GRO-Seq we provide the first genome-wide analysis of the nascent RNAs in iPSCs, HLCs and primary hepatocytes to extend our understanding of the transcriptional changes occurring during hepatic differentiation process. We demonstrate that a large fraction of hepatocyte-specific genes are regulated at transcriptional level and identify hundreds of differentially expressed non-coding RNAs (ncRNAs), including primary miRNAs (pri-miRNAs) and long non-coding RNAs (lncRNAs). Differentiation induced alternative transcription start site (TSS) usage between the cell types as evidenced for miR-221/222 and miR-3613/15a/16-1 clusters. We demonstrate that lncRNAs and coding genes are tightly co-expressed and could thus be co-regulated. Finally, we identified sets of transcriptional regulators that might drive transcriptional changes during hepatocyte differentiation. These included RARG, E2F1, SP1 and FOXH1, which were associated with the down-regulated transcripts, and hepatocyte-specific TFs such as FOXA1, FOXA2, HNF1B, HNF4A and CEBPA, as well as RXR, PPAR, AP-1, JUNB, JUND and BATF, which were associated with up-regulated transcripts. In summary, this study clarifies the role of regulatory ncRNAs and TFs in differentiation of HLCs from iPSCs.

## Introduction

Primary human hepatocytes (PHH) are extensively needed for both basic and translational science but their use is restricted by the lack of donors, and the limited number of cells since functional PHHs cannot be expanded *in vitro* and they rapidly lose their functionality during culture^1^. Stem cells offer an alternative, as they possess the ability to self-replicate and differentiate into all cell types in the body. Several protocols have been published for the production of hepatocyte-like cells (HLCs) from human embryonic stem cells and human induced pluripotent stem cells (iPSCs) through a definitive endoderm stage^2^. Producing hepatocytes from iPSCs offers a way to study different aspects of hepatocyte function in a patient-specific cell model, thus in the context of the genetic background of the patient.

Transcription constitutes the first step of gene expression and hence a fundamental cellular function. Microarrays have widely been replaced by massively parallel sequencing methods like RNA-sequencing (RNA-seq) as a method of studying whole transcriptomes (known and novel transcripts)^3^. RNA-seq enriches for stable mRNAs, which accumulate to steady-state levels in cells whereas global run-on sequencing (GRO-seq) measures production rates of primary transcripts by directly measuring nascent RNA production^4^. Hence, GRO-seq identifies the genes that *are being transcribed* at a given time point in a cell. It provides extensive information on the location and quantity of coding and non-coding transcripts, including promoter- and enhancer associated long non-coding RNAs (lncRNAs) and primary microRNAs (pri-miRNAs^5,6^). Furthermore, GRO-seq signal being independent of the stability of the transcripts produced, captures the correlation between gene transcription and enhancer activity and can be used to infer important transcription factors (TFs) based on enrichment of TF motifs^7,8^.

In this study, we generated patient-derived iPSCs and differentiated them into HLCs using protocols derived from two widely used methods previously described by Si-Tayeb^9^ (M1) and Hay^10^ (M2). We then performed GRO-seq to investigate the nascent transcription in the HLCs to gain better understanding of gene regulatory processes controlling hepatic differentiation. We were specifically interested in identifying active regulatory RNAs such as pri-miRNAs and lncRNAs that could contribute to the differentiation of functional HLCs from iPSCs.

## Results

### Characterization of HLCs

HLCs were generated from patient-derived iPSCs using two protocols derived from the ones described by Si-Tayeb *et al*.^9^ (Method 1, M1) and Hay *et al*.^10^ (Method 2, M2), with minor modifications (see Supplementary methods for details). During the first stage (days 1–6) the cells were directed from iPSCs into definitive endoderm cells, and then towards HLCs (Supplementary Fig. S1A,B). The qPCR analysis showed high expression of a pluripotency marker Oct3/4 at days 0–2 followed by upregulation of the definitive endoderm genes SOX17 and FOXA2 at days 5–8 (Supplementary Fig. S1C,D). The hepatic marker genes AFP and ALB were highly upregulated at day 21 (M1) and day 19–20 (M2), LDL-receptor (LDLR) gene was expressed throughout the differentiation (Supplementary Fig. S1C,D). Uniform expression of AFP protein was seen at day 20 whereas the expression of ALB was more sporadic in the culture (Supplementary Fig. S2A,B). The immunocytochemical analysis showed that LDLR is widely expressed also at protein level at day 21 (Supplementary Fig. S2C). Similarly, asialoglycoprotein receptor 1 (ASGR1) was expressed throughout the culture (Supplementary Fig. S2D). The HLCs were able to store lipids as shown by Oil red O staining, and produce albumin and urea but the levels were statistically significantly lower than for PHHs (Supplementary Fig. S2E–F). M1-HLCs produced TG at similar levels to PHHs whereas M2-HLCs produced less TG than the PHHs (Supplementary Fig. S2F).

### Gene expression profiling by GRO-seq

Protein-coding genes comprised most of the transcripts detected in all sample groups, as expected and ncRNAs such as pri-miRNAs and lncRNAs were the second prevalent transcript type (Supplementary Fig. S2G).

#### Protein-coding genes

**Figure 1:**
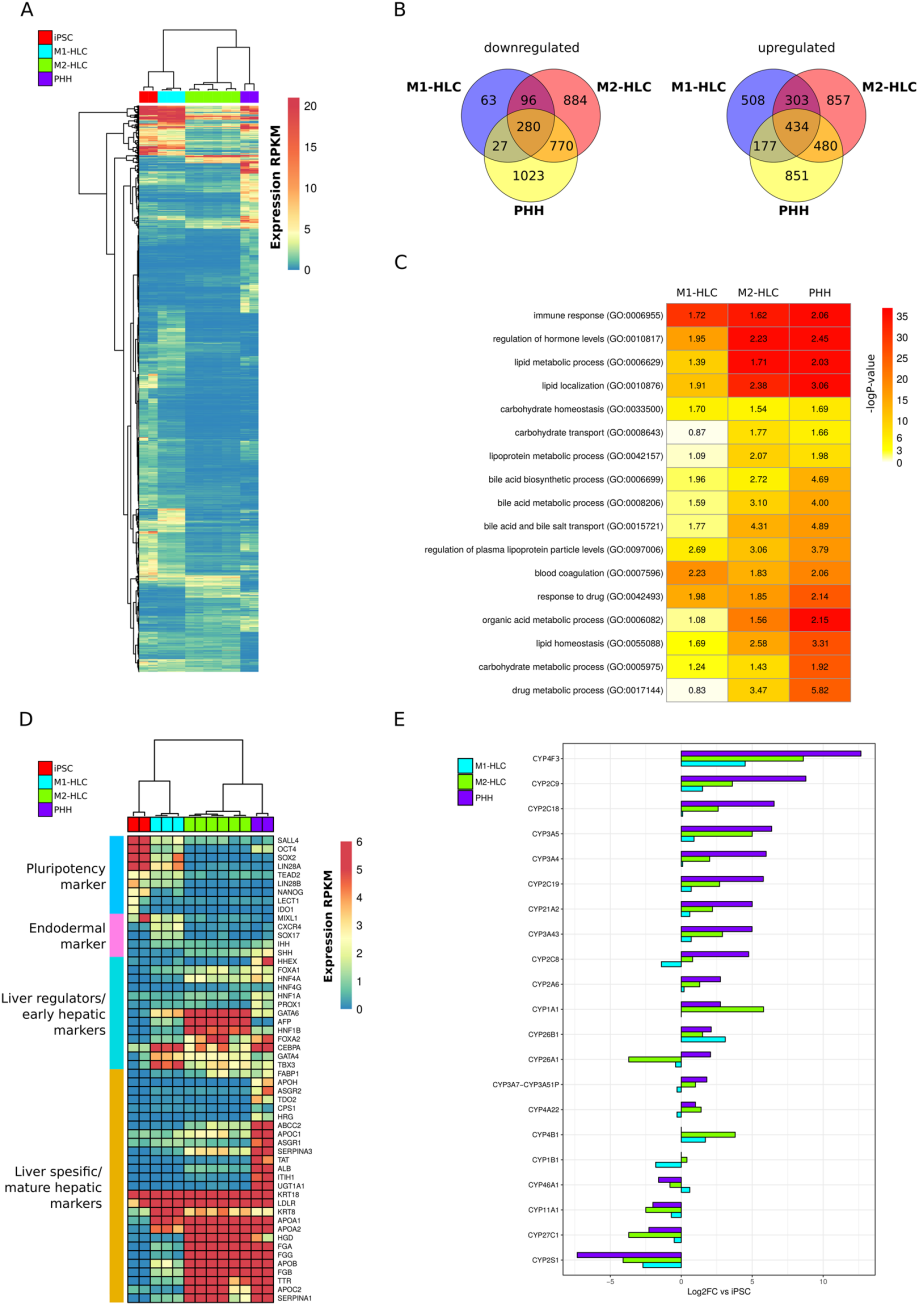
Protein-coding transcripts in estimating the hepatic differentiation. (**A**) Heatmap representation of the protein-coding genes in iPSCs, PHHs, M1- and M2-HLCs (FPKM > 1 in at least one of the samples; logFC > 2 and adj. p-value < 0.05 for at least one comparison). Unsupervised hierarchical cluster analysis was performed on differentially expressed protein-coding genes between iPSCs, PHHs and HLCs. A dendrogram demonstrates similarity and heatmap illustrates gene expression changes between the samples. Different samples are listed in columns and genes in rows. (**B**) Venn diagrams of differentially expressed protein-coding genes when comparing PHHs/M1-HLCs/M2-HLCs to iPSCs. (**C**) Gene ontology (GO) analyses of the differentially expressed protein-coding genes in M1-HLCs, M2-HLCs and PHHs vs. iPSCs. (**D**) Heatmap representation of a restricted set of genes related to pluripotency, hepatic differentiation and liver functions. (**E**) A bar chart representation of 21 cytochrome P450 (CYP) enzymes that were differentially expressed between the M1- and M2-HLCs (adj. p-value < 0.05). See also Supplementary Fig. S5.

Heatmap representation of the protein-coding genes illustrates the differentially expressed genes between the sample groups (Fig. 1A). In total 1942 up- and 2100 down-regulated protein-coding genes were identified when comparing the PHHs to iPSCs (Fig. 1B). To gain insight on which key biological pathways are active in PHHs, a gene ontology (GO) enrichment analysis was conducted for the differentially expressed genes (DEGs), separately for the sets of up- (Supplementary Table S1) and down-regulated protein-coding genes (Supplementary Table S2) in PHHs *vs*. iPSCs. For up-regulated genes, the most enriched terms were drug metabolic process, bile acid related processes and regulation of plasma lipoprotein levels (Fig. 1C). Furthermore, other liver-related biological processes such as organic acid metabolism, several lipid-related processes, regulation of hormone levels and blood coagulation were enriched. The down-regulated genes in PHHs (i.e. up-regulated in iPSCs) were enriched for GO terms related to e.g. stem cell proliferation and differentiation. When comparing the expression profiles of M1- and M2-HLCs to the iPSCs, in total 1422/2074 up- and 466/2030 down-regulated protein-coding genes were observed in M1/M2-HLCs, respectively (Fig. 1B). The up-regulated genes in M1/M2-HLCs (vs. iPSCs) shared enrichment of several GO terms with PHHs. Specifically, the drug metabolic process, regulation of hormone levels, lipid/lipoprotein and bile acid related pathways were enriched in M2-HLCs and PHHs, and immune response, regulation of plasma lipoprotein particle levels, blood coagulation and response to drug in M1-HLCs and PHHs (Fig. 1C).

#### Analysis on restricted set of pluripotency and liver-specific genes

Several known pluripotency TFs such as SALL4, OCT4, SOX2, LIN28, NANOG and TEAD2 had highest expression levels in the iPSCs (Fig. 1D) whereas liver-specific genes like APOH, CPS1, ASGR1, ALB and ITIH1 were statistically significantly up-regulated in PHHs compared with iPSCs. In the M1- and M2-HLCs, the gene expression levels of the plasma proteins (e.g. FGA, FGB, APOA2, APOB), liver-related genes (ALB, AFP, TTR) and several key hepatic TFs such as HNF4A, HNF1B, HNF1A, FOXA1 and FOXA2 were well in concordance with other published studies with similar differentiation protocols and total liver expression profiles (Supplementary Figs S3 and S4 and Supplementary Table S3).

#### Drug-metabolizing enzyme and transporter gene expression

In order for the HLCs to be usable in drug toxicity and efficacy testing, they need to express genes encoding drug-metabolizing enzymes, especially cytochrome P450 (CYP) enzymes. In total, we were able to measure the expression of 55 different CYP enzyme genes (Supplementary Table S4). The CYP3A5 was the highest expressed family member in PHHs and M2-HLCs. For most CYP-members, the expression was highest in PHHs, with the exception of CYP1A1 exhibiting higher expression in M2-HLCs. (Fig. 1E and Supplementary Fig. S5A,B). Despite this, CYP4F3, CYP2E1, CYP8B1 and CYP27A1 were statistically significantly more highly expressed in HLCs compared to iPSCs (log2FC > 1, p < 0,05; Supplementary Fig. S5A and Table S4). Moreover, we detected expression of 19 UDP-glucuronosyltransferases (UGT) and three UDP-glycosyltransferases. The nine detected enzymes of the UGT1A family were expressed at very constant levels within each sample group (Supplementary Fig. S6A). Levels of the UGT2A and UGT2B enzymes were highest for the PHHs, with the exception of UGT2B11, which was equally expressed in PHH and M2-HLCs, and UGT2B28, which was statistically significantly higher in M2-HLCs than in any other group (Supplementary Fig. S6B and Supplementary Table S4). Expression of different transporter genes varied more, highest expression was detected for ABCC2 and ABCC3 in the PHHs and for SLCO2B1 and SLCO10A1 in the M2-HLCs (Supplementary Fig. S6C,D).

#### Primary miRNA (pri-miRNA) expression and TSS switching

**Figure 2:**
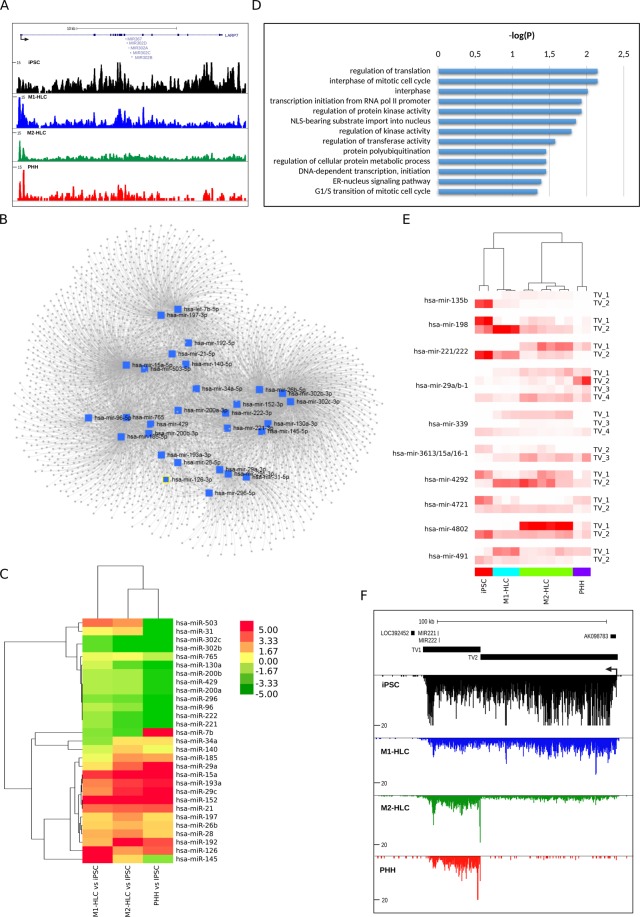
Identification of differentially expressed pri-miRNA transcripts and alternative TSS usage at pri-miRNA loci. (**A**) UCSC Genome browser shot image depicting normalized GRO-seq taq counts for the miR-302–367 cluster pri-miRNAs. (**B**) A pri-miRNA network of 29 hub miRNAs identified through miRNET tool, which was used to construct liver-specific miRNA target networks connecting miRNA to genes that had been validated by CLIP-studies. (**C**) A heatmap representation of the 29 hub miRNAs. Scale bar represents log2 fold change of M1-HLC/M2-HLC/PHH vs iPSC. (**D**) GO analysis of the hub miRNA target genes. (**E**) Quantification of differential transcription start site (TSS) activity at intergenic pri-miRNA loci with multiple TSSs. The differential TSS activity is calculated based on the GRO-seq signal difference between adjacent elements (TV FPKM = TV − TV + 1 FPKM). Pri-miRNAs were clustered using pairwise average linkage with un-centered correlation distance. (**F**) GRO-seq signal across the four cell types at the miR-221 locus, where two distinct TVs were identified. TV = transcript variant. See also Supplementary Fig. S2.

By using the previously annotated pri-miRNA coordinates^6^, we demonstrate that 563 pri-miRNAs were expressed in at least one of our sample groups. In total 253 pri-miRNAs were differentially expressed between the PHHs and iPSCs (Supplementary Table S5). The statistically significantly up-regulated pri-miRNAs in iPSCs vs. PHH included the well-established pluripotency-associated miRNA clusters like miR-302–367 (miR-302a/b/c/d/-367; Fig. 2A), miR-106a-363 (miR-106a/-18b/-19b-2/-20b/-363/92a-2), miR-106b-25 (miR-106b/-25/-93) and miR-17–92 (miR-17/-18a/19a/19b-1/20a/-92a-1), which were also more highly expresssed in M1- compared to M2-HLCs. The 99 miRNA clusters that were up-regulated in the PHHs vs. iPSCs included clusters like miR-29b-2/-29c, let-7a-3 (let-7a-3/-7b/miR-3619/-4763), miR-29a/-29b-1, miR-193a/365b/4725 and miR-192/-194-2 (Supplementary Table S5).

Next, we used mirNET tool^11^ to construct liver-specific miRNA target networks connecting miRNA to genes that had been validated by CLIP-studies (CLIP = Crosslinking and immunoprecipitation). This analysis identified 29 hub miRNAs that could regulate ~3000 target mRNAs (Fig. 2B). The hub miRNAs included pluripotency miRNAs miR-302b and -302c, and liver-related miRNAs like miR-21, miR-192 and miR-34a. Similar to protein coding genes, the expression of the hub miRNAs clustered the M2-HLCs and PHHs closer together than the M1-HLCs and PHHs (Fig. 2C), and the GO analysis indicated that the hub miRNA target genes were mostly related to regulation of translation, initiation of transcription, regulation of protein metabolism as well as cell cycle (Fig. 2D). MiR-34a, -29a, -29c and -192 exhibited an increasing expression in the order M1- < M2-HLCs < PHHs (Fig. 2C). To validate the GRO-seq results, expression of eight pri-miRNAs was measured at mature miRNA level by RT-qPCR in M1- and M2-HLCs, as well as in PHHs and human liver (hTLR). In line with the GRO-seq results, miR-302b, miR-9*, miR-221-3p and miR-222-3p expression was highest in the iPSCs. On the other hand, miR-21-5p and miR-122 expression was higher in the HLCs, PHHs and hTLR. Expression of miR-29a-3p and miR-15a-5p was highest in PHHs and hTLR, and higher in M2- than M1-HLCs. (Supplementary Fig. S2H).

Transcriptional profiling of pri-miRNAs allowed us to evaluate the contribution of different transcript variants (TVs) originating from distinct TSSs. We detected in total 78 differentially expressed TVs with 2 to 6 alternative TSSs between the groups (Supplementary Table S6). The most pronounced effect of differentiation in TSS switching was seen for miR-135b, -198, -221/222, -29a/b-1, -339, -3613/15a/16-1, -4292, -4721, -4802 and -491 (Fig. 2E). Interestingly, the miR-29a/b-1 TV4, which shares the TSS with lncRNA LINC-PINT, had highest expression in M2-HLCs whereas the PHHs expressed more TV2. The two TSSs of miR-221/222 had differential activity across the studied cell types: TV1 was highly expressed in M2-HLC and PHHs whereas TV2 activity was higher for M1-HLCs and iPSCs (Fig. 2F and Supplementary Table S6). In addition, miR-3613/15a/16-1 showed alternative TSS usage: TV1 was more active in iPSCs and TV2 in HLCs and PHHs. Overall, hepatocyte differentiation associated with extensive changes in pri-miRNA expression, which in some cases involved alternative TSS usage.

#### HLC differentiation is encompassed by extensive changes in lncRNAs expression

**Figure 3:**
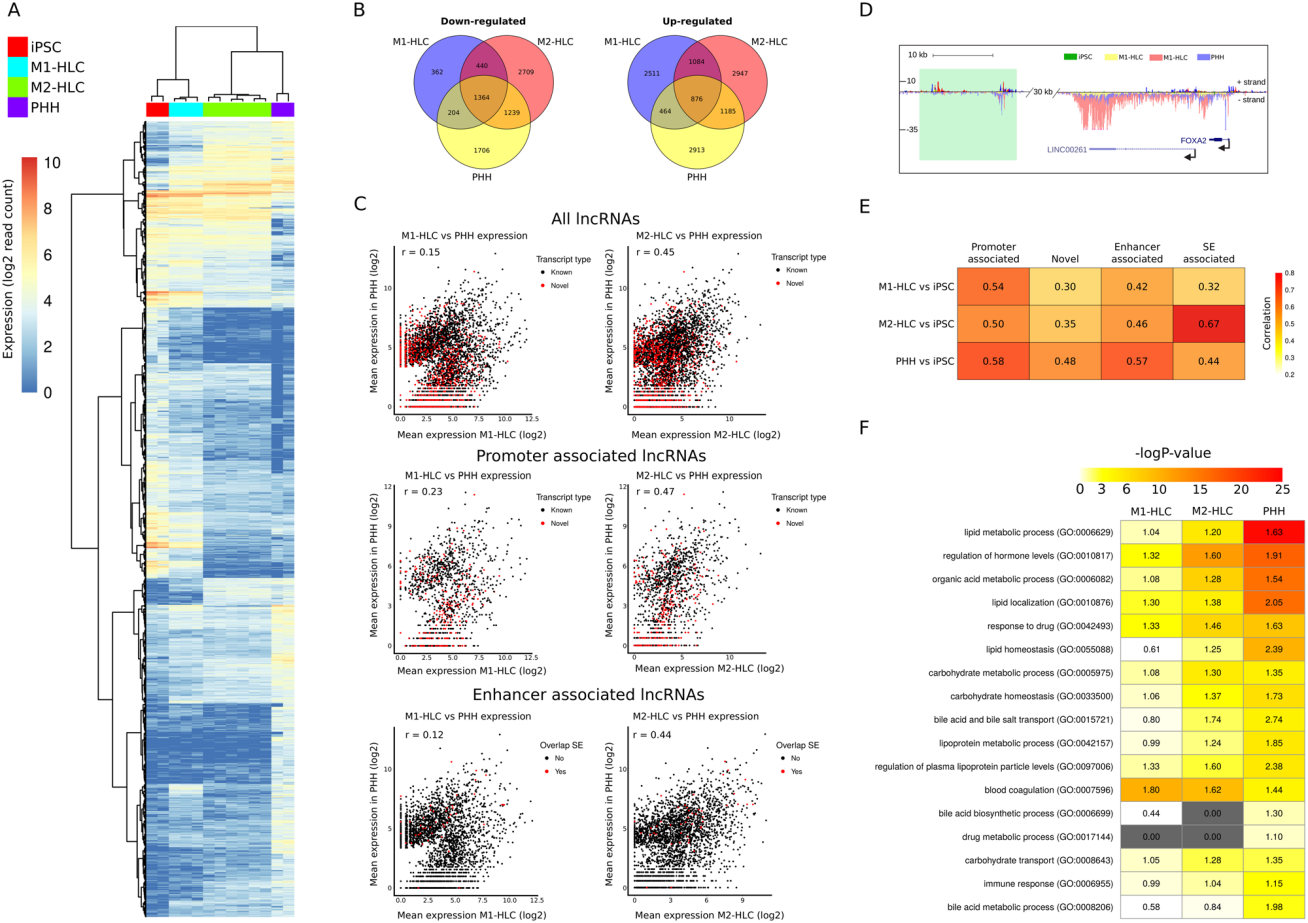
HLC differentiation is encompassed by extensive changes in lncRNAs expression. (**A**) Heatmap representation of the differentially expressed (log2FC > 2 and adj. p-value ≤ 0.05) lncRNAs. Samples are listed in columns and genes in rows. (**B**) Venn diagram of the differentially expressed lncRNAs when comparing PHHs, M1- and M2-HLCs to iPSCs. (**C**) Correlation of the lncRNA expression in PHHs with M1- or M2-HLCs calculated separately for all lncRNAs, promoter- and enhancer-associated lncRNAs. Known and novel lncRNAs are marked with black and red, respectively. For enhancer-associated lncRNAs, the ones overlapping with super-enhancers (SE) are marked with red. (**D**) USCS genome browser image for the SE (marked in green) at the FOXA2 locus. (**E**) Correlation of differentially expressed lncRNAs with the nearby coding gene. lncRNAs were separated into novel, promoter-, enhancer- and SE-associated. Numbers within the boxes indicate the correlation coefficient, as does the color bar. (**F**) Enrichment of GO terms in the differentially expressed lncRNAs. Numbers state the enrichment factor and color indicates the −log2 p-value for each GO term according to the color bar.

In total 145 094 putative lncRNAs were discovered across the sample groups; 93.5% of these were previously annotated (LNCipedia 5.0 or RefSeq). We identified 9496 *novel* lncRNAs: 1531 bidirectional and 7965 unidirectional transcripts (Supplementary Table S7). The clustering of differentially expressed lncRNAs, known or novel, effectively separate the iPSC and M1 from the PHH and M2 samples similarly as DEGs (Fig. 3A). In line with this, pairwise comparison showed that M2-HLCs and PHHs share more differentially expressed lncRNAs than M1-HLCs and PHHs (Fig. 3B). Among the top up-regulated lncRNAs in the iPSCs were pluripotency-associated lncRNAs such as TUNAR, LINC00458 and LINC-ROR (*r*egulator *o*f *r*eprogramming). Their expression decreased statistically significantly during the iPSC to HLC differentiation by both methods but stayed at higher level with M1- than with M2-HLCs. Expression of some lncRNAs increased during the differentiation regardless of the method, including lnc-FOXA2-3:6, lnc-KLF6–17:1/-21:1, lnc-APOB-1:2 and LINC01595:2, for which the nearest genes were important hepatocyte-regulators FOXA2, KLF6, APOB and SULT2A1, respectively. Few lncRNAs were only induced in M1-HLCs as exemplified by BI758 and lnc-NOVA1–3, located close to IRS1 and NOVA1 genes, respectively. On the other hand, lnc-APOB-2:2, lnc-MUC4-1:1, lnc-GCLC-1:4 and UNI6253, located close APOB, MUC20, GLCL and PTP4A1 genes, respectively, were specifically upregulated in M2-HLCs (Supplementary Table S7).

To further categorize the lncRNAs, we divided them into promoter- (36%) and enhancer-associated (64%) (Supplementary Table S7) based on the prominent presence of H3K4me3 at the promoters and H3K4me1 at the enhancers^12^. Plotting the expression of differentially regulated lncRNAs (identified in PHHs vs. iPSCs) for M1- and M2-HLCs, demonstrated again higher correlation between M2-HLCs and PHH compared to M1-HLCs and PHH, for both promoter- and enhancer associated lncRNAs (Fig. 3C). Interestingly, 534 of the lncRNAs overlapped with hepatocyte-specific super-enhancers [SEs;^13^], majority of which were induced in differentiated HLCs. In line with previous findings, these SEs were found in close proximity to genes important for hepatocyte function and transcriptional control including FOXA2 (Fig. 3D)^13,14^.

Finally, we applied the guilt-by-association principle to infer functions of the identified lncRNAs. This approach is based on a correlation analysis between lncRNA and protein-coding gene expression in combination with the GO enrichment analysis of the protein-coding mRNA^15^. Altogether, a good correlation was detected between lncRNA and adjacent coding gene expression, irrespective of the lncRNA category (Fig. 3E). The pattern of GO term enrichment in the lncRNA target genes was similar to the pattern detected in protein-coding genes. The highest enrichment was detected for e.g. the lipid homeostasis, regulation of plasma lipoprotein particle levels, bile acid and salt transport, blood coagulation and response to drug (Fig. 3F). On the contrary, the ‘drug metabolic process’ was not detected in the HLC lncRNAs target genes and the enrichment was low even in the PHH lncRNAs target genes (Fig. 3F). This is opposite to protein-coding genes where ‘drug metabolic process’ was the most enriched process in the PHHs and highly enriched in the M2-HLCs (Fig. 1C).

### Changes in regulatory motifs reflect the altered repertoire of transcription factors (TFs)

**Figure 4:**
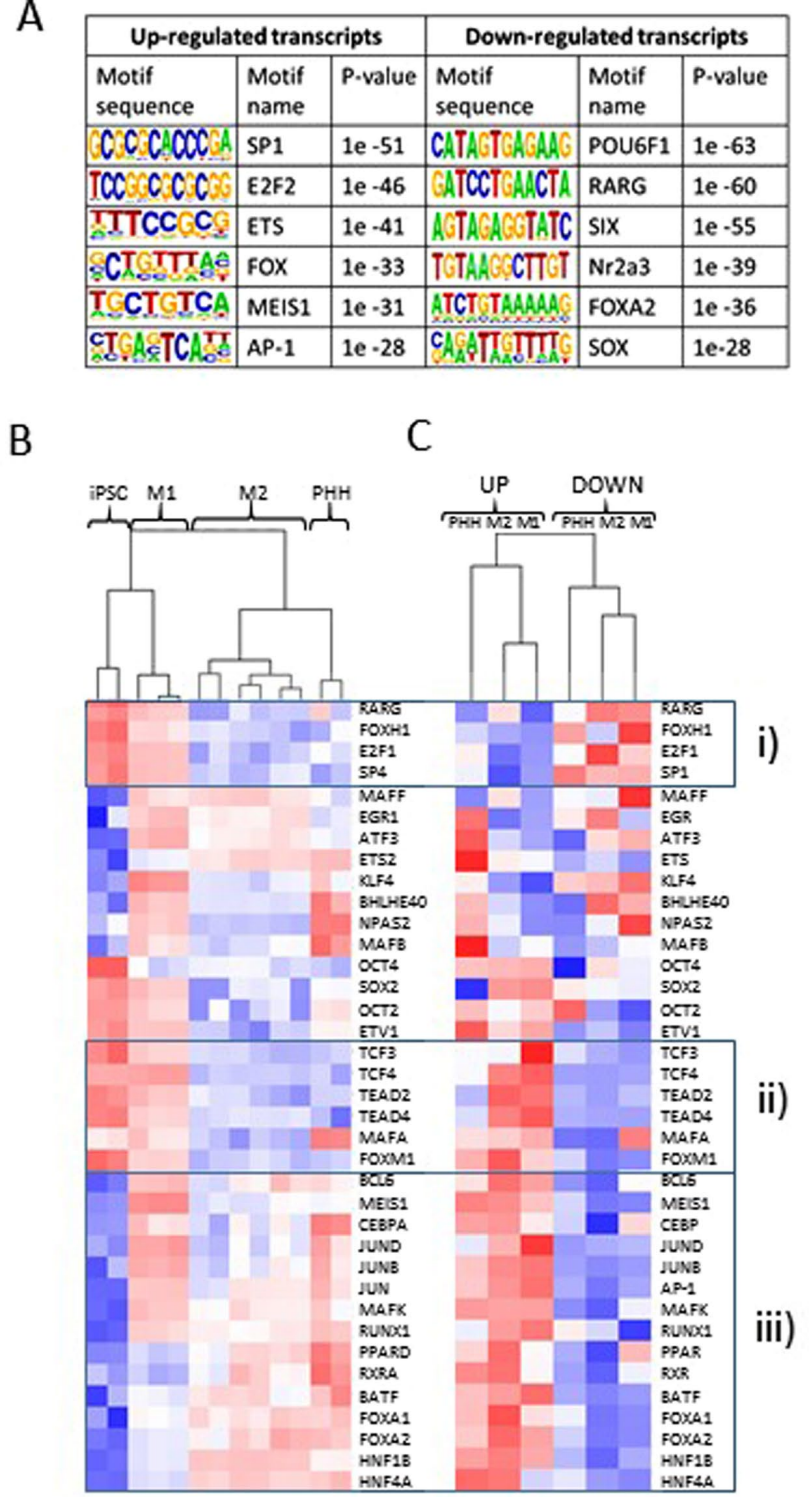
Changes in regulatory motifs reflect the altered repertoire of transcription factors (TFs). (**A**) Sequence motifs associated with lncRNAs for up- and down-regulated transcripts in PHHs vs. iPSCs. (**B**) Heatmap of normalized RPKM values (−1 to 1) of 32 differentially expressed TFs during hepatocyte differentiation for which motif information existed. Clustering was performed for genes and samples using Spearman’s rank correlation (complete linkage). (**C**) Heatmap of normalized motif enrichment percentages (−1 to 1) of the TFs listed in (**B**). (i) The down-regulated transcripts being enriched for potential transcriptional repressors, (ii) the up-regulated transcripts enriched for potential transcriptional repressors, (iii) the up-regulated lncRNAs transcripts being enriched for the binding of several hepatocyte-specific TFs.

Majority of binding sites for lineage-specific TFs locate at inter- and intragenic enhancer regions, outside the promoters of coding genes^16,17^. Recent studies have further demonstrated that highly active enhancers display H3K4me3-promoter mark^18^ but also that promoters can serve as enhancers^19,20^. This prompted us to focus the identification of regulator TF binding sites to the TSSs of all lncRNAs identified above. The top TF motifs found enriched at up-regulated TSSs were SP1, E2F2 and ETS whereas the down-regulated (in PHHs vs. iPSCs) TSSs were enriched for POU6F1, RARG and SIX motifs (Fig. 4A). This suggests that a subset of TFs that exhibit significant changes in response to differentiation could be responsible for establishing the hepatocyte-specific transcriptional profiles. Altogether, we identified 137 TFs statistically significantly up- and 174 down-regulated in PHHs vs. iPSCs (Supplementary Table S8). Among the most up-regulated TFs were liver-related HNF4A, HNF1B, FOXA1 and FOXA2^21^, which were also up-regulated in the HLCs but remained statistically significantly lower in the M1- than M2-HLCs. Among the most down-regulated TFs were pluripotency-related FOXD3^22^, ZIC3, SOX2 and NANOG^23^, as well as SOX11^21^ and OTX2^24^. These were all down-regulated during the HLC differentiation but remained statistically significantly higher in the M1- than M2-HLCs, in which the expression reached the levels detected in the PHHs (Supplementary Table S8).

To study the contribution of the TFs to transcriptional programs, we correlated the TF expression levels (of TFs for which motifs have been well established) with the relative enrichment of the motif at regulated lncRNA TSSs (Fig. 4B,C). Our analysis identified several subgroups: (i) the down-regulated transcripts being enriched for the binding of pluripotency and cell cycle associated TFs such as RARG, E2F1, SP1 and FOXH1; (ii) the up-regulated transcripts being enriched for potential transcriptional repressors, whose expression is dampened in hepatocytes exemplified by TEAD2, TEAD4, TCF3, TCF4 and FOXM1; (iii) the up-regulated lncRNA transcripts being enriched for the binding of hepatocyte-specific TFs such as FOXA1, FOXA2, HNF1B, HNF4A and CEBPA; as well as AP-1 along with its subunits/family members JUNB, JUND and BATF (Fig. 4C). Also, the motifs of RXR and PPAR were enriched in the up-regulated lncRNAs transcripts.

## Discussion

In the present study, we produced iPSCs from patient-derived fibroblasts, and differentiated them into hepatocyte-like cells (HLCs) by two different methods modified from the protocols originally described by Si-Tayeb^9^; [referred to as M1] and Hay^10^; [referred to as M2]. Moreover, we measured nascent RNA transcription in iPSCs, HLCs and primary human hepatocytes (PHHs) by GRO-sequencing^4^, which provides a “map” of the position and orientation of all engaged RNA polymerases across the genome at extremely high resolution. We demonstrate, for the first time, extensive changes in the nascent transcriptional profiles during the iPSC to HLC differentiation, concurrently delineating the expression profile of modulatory ncRNAs like miRNAs and lncRNAs.

Maturation of HLCs remains a challenge as they continue to express AFP, which is a marker of a fetal, immature hepatocyte phenotype. Despite the persisting AFP positivity, the iPSC-derived HLCs possessed hepatocyte functionality, which was confirmed by albumin, urea and triglyceride secretion. Extensive analysis of nascent coding and non-coding RNA expression provided further evidence on successful hepatic differentiation by M1 and M2 methods. Through pathway analyses, we further demonstrated an enrichment of liver-related GO terms in both M1- and M2-HLCs but the enrichment remained lower than in PHHs. Recently these differentiation protocols have been updated and new methods developed^25–28^, and future studies are needed to identify the optimal protocol. However, our study provides several candidate genes among the coding and non-coding RNAs that could be used as markers to evaluate the efficiency of differentiation.

To be usable as an *in vitro* model for studying human hepatic drug metabolism, transport, clearance and toxicity, the HLCs have to express a set of enzymes and transporters involved in hepatic drug clearance. The most important drug-metabolizing enzymes belong to the cytochrome P450 (CYP) superfamily. Overall, the CYP gene expression levels were lower in the HLCs than the PHHs. This was in line with the GO enrichment analyses showing that drug metabolic process was highly enriched in the PHHs but less in the HLCs. The expression of CYP3A family is of interest as it, especially CYP3A4, is responsible for the metabolism of majority of drugs and highly expressed in the liver^29,30^. CYP3A4 was highly expressed in the PHHs and statistically significantly more in M2- than M1-HLCs. Another major isoenzyme in human hepatocytes is CYP1A2^31^, which was expressed only at very low levels in the HLCs. The CYP1A1 level, on the other hand, was highest in M2-HLCs similarly as in a previous study detecting higher CYP1A1 than CYP1A2 levels in ESC- and iPSC-derived hepatocytes^32^. High CYP1A1 expression in M2-HLCs speaks against the cells being of fetal phenotype as fetal hepatocytes express very low levels of this CYP enzyme^33^. In conclusion, regardless of the large variety of CYP enzymes expressed in the HLCs, the PHHs still appear superior. However, we must acknowledge the limitation that our analysis is based on transcriptional level and further studies are needed to compare the differences in the drug metabolizing capacities of M1- and M2-HLCs compared to PHHs.

The abundance of pri-miRNAs is low at steady state and hence, they are poorly represented in standard RNA-seq libraries. GRO-seq measures the nascent transcriptome, thus enabling the analysis of active transcription at miRNA loci. This allowed us to detect the expression of 564 pri-miRNAs of which 45% were differentially regulated. The expression of pri-miRNA clusters, like the miR-302–367 and miR-17–92, encoding for pluripotency and proliferation-promoting miRNAs^34^ was highest in the iPSCs, with a gradient of declining expression in M1- > M2-HLCs > PHHs. Pluripotency TFs OCT4, SOX2 and Nanog are known upstream regulators of the miR-302 cluster^35^, which subsequently regulates cell cycle through cyclin D1 and Cdk4^36^. The cell cycle dynamics has a crucial impact on the differentiation potential of stem cells with longer G1 phase inducing differentiation in ESCs^37^. The important role of differentially expressed miRNAs in the regulation of cell cycle was further supported by the established role of many hub miRNAs, such as miR-21^38^, miR-15a^39^, miR-34a^40^, -29a^41^, -29c^42^ and -192^43^, and the miRNA target gene functions in cell cycle. Consequently, through regulation of the cell cycle miRNAs may participate in regulating proliferation, which reduces during iPSC to hepatocyte differentiation^44^.

Multiple TSSs are an important regulatory feature at miRNA loci and alternative TSS usage plays an important role in transcriptional control^45^. Alternative TSS usage can affect gene expression and result in tissue-specific and temporally regulated expression^46^. We have earlier shown usage of multiple TSSs to be common within intergenic pri-miRNA genes^6^. Here we show that active TSS locations change in response to hepatocyte differentiation from progenitors, as exemplified by alternative TSS usage at the miR-221/222 and miR-3613/15a/16-1 loci. In conclusion, alternative pri-miRNA TSS usage can take part in orchestrating the intricately controlled expression of specific genes during the iPSC to HLC differentiation. However, future studies are needed to elucidate how changes in pri-miRNAs levels or TSS usage translate to changes at mature miRNA level, and further on to changes at miRNA target gene levels.

The lncRNAs have no coding potential but exhibit tissue-specific expression^47^. A recent study identified >58000 lncRNA genes^48^ but the function is validated for only a small fraction of them^49^. LncRNA are known to take part in epigenetic, transcriptional (as activators or repressors), and post-transcriptional regulation of gene expression, as well as regulate stem cell pluripotency and differentiation^50^. The high levels of lncRNA-ROR (regulator of reprogramming) in the iPSCs in our study coincides with previous studies showing lncRNA-ROR to promote pluripotency and stem cell survival^51^. Also other pluripotency-associated lncRNAs like TUNAR^52^ and LINC00458^53^ decreased during the iPSC to HLC differentiation. LINC00458 is highly expressed in the early stages of hepatic differentiation^54^, and it modulates pluripotency by physically interacting with SOX2^53^. TUNAR mediates the recruitment of RNA-binding proteins to SOX2, NANOG and FGF4 promoters, thus activating their transcription and promoting pluripotency^52^. Concurrently, the expression of these pluripotency markers decreased during differentiation. Based on the histone methylation marks, we deduced that >60% of the lncRNAs were enhancer-associated. We detected good correlation between the expression of lncRNAs and adjacent coding genes, irrespective of the lncRNA category, suggesting that lncRNA and coding genes could be tightly co-expressed and thus co-regulated. Interestingly, several up-regulated lncRNAs (in HLCs vs. iPSCs) were situated close to KLF6, which is vital for definitive endoderm formation during hepatocyte differentiation^55^. In addition, in PHH and M2-HLCs lnc-MUC4-1 was upregulated, the nearest gene of which is MUC20. The protein encoded by this gene, Mucin-20, interacts with Met and eventually attenuates ERK1/2 activation, which is important for cell cycle regulation as well as hepatocyte survival^56^. Interestingly, inhibition of the ERK pathway has been shown to extend the life span of hepatocytes in culture as well as increase hepatocyte differentiation^57^. Thus, the lncRNAs could play an important part in the hepatocyte differentiation through regulating e.g. endoderm formation and cell cycle as well as liver-related functions in the differentiated HLCs. The molecular mechanisms behind this regulation need to be explored in further studies.

Correlation of the TF expression with the relative enrichment of the motifs at regulated lncRNA TSSs allowed us to identify a distinct set of transcriptional regulators that could participate in orchestrating the transcriptional changes in response to differentiation. Motifs of TFs like RARG, E2F1, SP1 and FOXH1, whose expression was repressed in PHHs and M2-HLCs, were enriched in the down-regulated transcripts in all the hepatocytes. For example, RARG activates endogenous OCT4 expression and is thus important for somatic cell reprogramming to iPSCs^58^. On the other hand, E2F1 transcription factor is involved in regulating the cell cycle^59^ again suggesting that modulating the cell cycle progression is vital for successful HLC differentiation. FOXH1 mainly acts as a transcriptional activator and could be pivotal for the formation of mesendoderm^60^, the major precursor of definitive endoderm^61^. Its higher levels in M1- compared to M2-HLCs suggests that endodermal features persist in M1-HLCs. Motifs of transcriptional regulators like TEAD2, TEAD4, TCF3, TCF4 and FOXM1, whose expression was repressed in PHHs and M2-HLCs, were enriched in the up-regulated transcripts suggesting that the main function of these factors was to repress gene expression in progenitor cells. The TEAD1-4 proteins interact with YAP to form functional heterodimeric TFs that act in the Hippo signaling pathway regulating cell proliferation, differentiation and cell death^62^. TEAD2 has earlier been shown to be highly expressed in embryonic liver and dramatically downregulated in adult liver^63^. Thus, higher levels of TEAD2 in M1-HLCs suggests they could possess a more proliferative and embryonic phenotype than the M2-HLCs. Similarly, FOXM1, a pro-proliferative TF acting as a stimulator of cell cycle progression and expression of pluripotency genes^64^, had high expression in the iPSCs and higher in M1- compared to M2-HLCs. Finally, the upregulated lncRNA transcripts in PHHs and HLCs were enriched for the binding of hepatocyte-specific TFs such as FOXA1, FOXA2, HNF1B, HNF4A and CEBPA. Most of these TFs and their binding sites have previously been correlated with a set of genes that are enriched with mature liver functions^21^. HNF1B, HNF4A, FOXA1 and FOXA2 are vital for hepatocyte differentiation^65^, and they were all more induced in HLCs. Moreover, HNF4A is known to maintain hepatic expression of certain miRNAs like miR-192 and miR-194^66^, and thus play a key role in the indirect regulation of hepatic transcriptome. Interestingly, PPARD and RXR that are known to act as heterodimers^67^ and regulate genes important for cell differentiation as well as lipid and cholesterol metabolism^68,69^ were both up-regulated in PHH and M2-HLCs (vs. iPSC). Another up-regulated TF in PHH and M2-HLCs was BATF, which increases chromatin accessibility to allow subsequent binding by other TFs^70^. BATF plays an essential role in the differentiation and function of various effector T cell subsets by regulating expression of key TFs, cytokines as well as cytokine and chemokine receptors^71^. BATF acts as a pioneering factor by binding cooperatively with the TF IRF4 and its dimerization partners like JunB and JunD^70^. Our data suggests that it might also play a previously unrecognized role in the hepatocyte differentiation as the up-regulated lncRNA transcripts were highly enriched for the BATF motif as well as the motifs of its binding partners JUNB and JUND. This information could provide future means for the generation of more mature hepatocytes through modulation of TF expression.

## Conclusions

We present here the first study characterizing the nascent transcriptome of the iPSC-derived HLCs that expands the analysis from protein coding genes to lncRNAs and pri-miRNAs. We provide evidence that abundance of transcriptional regulators and miRNAs central to mature hepatocyte function are indeed regulated at transcriptional level. Moreover, we identified potential novel master regulators that could orchestrate the transcriptional changes during hepatocyte-specific differentiation. Altogether, this information could be used to improve the current hepatic differentiation protocols aiming at mature hepatocyte phenotypes.

## Material and Methods

### iPSC reprogramming and cell culture

The study was approved by the Ethical Committee of Pirkanmaa Hospital District (Ethical approval no R12123) and written informed consent was obtained from all fibroblast donors. The iPSC lines were produced from skin fibroblasts with Sendai virus vectors and maintained as published before^72^. All experiments were performed in accordance with relevant guidelines and regulations. Patients donating skin biopsies signed an informed consent after receiving both an oral and written description of the study. Altogether three iPSC lines (UTA.10100.EURCAs, UTA.11104.EURCAs, and UTA.11304.EURCCs) were used in this study, and the lines were characterized as previously described^73^.

### Differentiation of iPSCs into hepatocyte-like cells (HLCs)

To differentiate iPSCs to HLCs we used two methods derived from the ones described by Si-Tayeb *et al*.^9^ (Method 1, M1) and Hay *et al*.^10^ (Method 2, M2), both consisting of three main stages (Supplementary Fig. S1A). Details of the differentiation protocols and their modifications are described in the supplement.

### Primary human hepatocytes (PHH)

Commercially available primary human hepatocytes (PHH; Gibco, Invitrogen) from two different donors (Hu8209, male, Caucasian, 50 years and Hu8132, female, Caucasian, 57 years) were used as reference. The plateable cryopreserved PHHs were thawed and plated according to manufacturer’s instructions, and the cells collected for GRO-seq on the next day (within 24 h after plating).

### Real-time quantitative PCR (RT-qPCR) and immunocytochemistry

RNA extraction, cDNA synthesis and RT-qPCR was performed as described earlier^73^. The expression of mature miRNAs or mRNAs was verified by RT-qPCR by using the TaqMan miRNA assays, advanced miRNA assay or mRNA assays (assay details can be found in the supplementary methods). Cultured cells were fixed and stained as described before^73^.

### Functionality of the HLCs

Functionality of the differentiated HLCs was studied by measuring the LDL uptake, albumin, urea and triglyseride secretion of the cells. The lipid storing capability of HLCs was studied by Oil red O staining.

### Global Run-On sequencing (GRO-seq) and data analysis

The nuclear run-on and library preparation was performed as described in^74^. The filtered data was mapped using Bowtie^75^ and data analysis performed using HOMER 4.7.^16^. More detailed information on data analysis can be found in the supplement.

## Supplementary information

Supplementary Information

Supplementary Table S1

Supplementary Table S2

Supplementary Table S3

Supplementary Table S4

Supplementary Table S5

Supplementary Table S6

Supplementary Table S7

Supplementary Table S8

## Data Availability

HepG2 H3K4me1 (GSM798321) HepG2 H3K4me3 (GSM733737) H1-hESC H3K4me1 (GSM733657) H1-hESC H3K4me3 (GSM733782). The datasets generated during the current study are available in the NCBI Gene Expression Omnibus repository under accession number GSE117086.
