# Supplementary material for: Extensive reprogramming of the nascent transcriptome during iPSC to hepatocyte differentiation
Source: Sci Rep. 2019 Mar 5;9:3562. doi: 10.1038/s41598-019-39215-0 (PMC6401154; doi:10.1038/s41598-019-39215-0)
Supplement: Supplementary file 1 — Supplementary Information [file 41598_2019_39215_MOESM1_ESM.pdf]

Supplementary information

Extensive reprogramming of the nascent transcriptome during iPSC to hepatocyte differentiation

Leena E. Viiri[1*ø], Tommi Rantapero[2*], Mostafa Kiamehr[1], Anna Alexanova[1], Mikko Oittinen[3], Keijo Viiri[3], Henri Niskanen[4], Matti Nykter[2], Minna U. Kaikkonen[4§], Katriina Aalto-Setälä[1,5§]

*These authors contributed equally
ø Corresponding author
§These authors share senior authorship

[1] Finnish Cardiovascular Research Center Tampere, Faculty of Medicine and Health Technology, Tampere University, Tampere, 33014 Finland.

[2] Prostate Cancer Research Center, Faculty of Medicine and Health Technology, Tampere University, Tampere 33014 Finland.

[3] Tampere Center for Child Health Research, Faculty of Medicine and Health Technology, Tampere University, Tampere 33014 Finland.

[4] A. I. Virtanen Institute for Molecular Sciences, University of Eastern Finland, Kuopio 70211, Finland.

[5] Heart Center, Tampere University Hospital, Tampere 33520, Finland.

Contact: leena.viiri@staff.uta.fi

Supplementary Methods

Differentiation of iPSCs into hepatocyte-like cells (HLCs)

Before starting hepatic differentiation, patient-derived iPSCs were adapted to feeder-free conditions by plating them on Geltrex (Gibco®, 1:100 dilution) in mTeSR™ medium (STEMCELL™ Technologies, France). The iPSCs were cultured on Geltrex until the colonies became 60-70% confluent. In the first method (Method 1, M1; modified from [1]) differentiation was initiated by culturing the iPSCs for 5 days with 100ng/ml Activin A (PeptroTech) in RPMI1640+Glutamax medium (Gibco, Invitrogen), 2% B27, and adding 3µM CHIR99021 (StemGent) and 1mM NaB (Sigma-Aldrich) on the first day. On the second day, CHIR was left out and NaB was dropped to 0.5mM for the next 4 days. This was followed by 3 days with 20ng/ml BMP4 (R&D Systems), 10ng/ml basic fibroblast growth factor (bFGF; PeproTech) in RPMI1640+Glutamax, 2% B27; then 5 days with 20ng/ml HGF (Gibco, Invitrogen) in RPMI1640+Glutamax, 2% B27, and finally for 8-9 days with 50ng/ml HGF, 20ng/ml Oncostatin-M (R&D Systems) in Hepatocyte Culture Media (Lonza) supplemented with SingleQuots (without EGF) (Fig. S1A). As opposed to the original protocol, hypoxic incubator was not available, thus 20% oxygen level was used throughout. This is in line with previous studies using normoxic conditions [2,3] . Also, HGF was used in addition to OSM at the last stage of differentiation to promote hepatic maturation [4].

The second differentiation method (Method 2, M2; modified from [5]) was initiated by switching the iPSC culture medium to RPMI1640+GlutaMax medium (Gibco, Invitrogen) supplemented with 100ng/ml Activin A (PeproTech), 75 ng/ml Wnt3 (R&D Systems), 2% B27 (Gibco, Invitrogen) and 1mM NaB (Sigma-Aldrich) on the first day (0.5 mM NaB from day 2. To reach the definitive endoderm stage the cells were grown in this medium for 5-6 days and the medium was changed every day. When the cells had reached the definitive endoderm stage, hepatic differentiation was initiated by switching the medium to KO-DMEM+20% KSR, 1mM Glutamax, 1% NEAA, 0.1% 2-ME, and 1% Dimethyl sulfoxide (DMSO) for 7 days. At the last stage (~from day 15 onwards) the cells were cultured in Leibovitz's L-15 medium (Invitrogen), supplemented with 8.3% fetal bovine serum (Biosera), 8.3% Tryptose phosphate broth (Sigma-Aldrich), 10 µM Hydrocortisone 21-hemisuccinate, 1mM Insulin (both from Sigma-Aldrich), 25 ng/ml Hepatocyte growth factor (HGF; Invitrogen), and 20 ng/ml Oncostatin M (R&D systems) and 0.5% penicillin/streptomycin. (Fig. S1A). Slightly higher Wnt3a and HGF concentrations were used compared to the original protocol to promote more efficient DE formation [6] and hepatic maturation [4], respectively.

Quantitative real-time qPCR

RNA samples were collected at days 0, 2, 5, 8, 13, 16 and 21 for M1 and at days 0, 2, 5 or 6 and 19 or 20 for M2 differentiations. RNA extraction, cDNA synthesis and RT-qPCR was performed as described earlier [7]. We verified the expression of four mature miRNAs by RT-qPCR by using the TaqMan miRNA assays (miR-9, ID 002231; miR-302b, ID 000531; miR-21, ID 000379; miR-122, ID 002245) and advanced miRNA assays (miR-29a-3p, ID 478587; miR-221-3p, ID 477981; miR-222-3p, ID 477982; miR-21-5p, ID477975; miR-15a-5p, ID477858; miR-302b-3p, ID 478591) . The U6 snRNA (ID 001973, for miRNA assays) or miR-186-5p (ID 477940; for advanced miRNA assays) was used as a reference gene for normalisation of target miRNA transcript levels. The expression of pluripotency and hepatic genes was validated by using the TaqMan mRNA assays (OCT3/4, ID Hs00999634_m1; AFP, ID Hs00173490_m1; ALB, Hs00609411_m1; APOA, ID, Hs00985000_g1; APOA2, Hs00155788_m1; APOB, Hs00181142_m1; FOXA2, Hs00232764_m1, ASGR1, Hs00155881_m1; FGA, Hs00241027_m1; HNF1B, Hs01001602_m1; HNF4A, Hs00604435_m1; SERPINA1, Hs00165475_m1; SERPINA3, Hs00153674_m1, CEBPA, Hs00269972_s1; CEBPB, Hs00270923_s1; CYP4F3, Hs01587865_g1; CYP3A5, Hs00241417_m1; CYP3A4, Hs00430021_m1 and CYP1A1, Hs00153120_m1. Each PCR reaction was run in triplicate and the ΔΔCt method was applied using iPSCs or the PHH as the reference group. First Choice® Human Liver Total RNA (hLTR, Cat. No. AM7960), purchased from Ambion®, was used as an extra control.

Functionality of the HLCs

LDL uptake of the HLCs was measured by using the LDL Uptake Assay Kit (Cayman Chemicals). Human Albumin ELISA Quantitation set (Bethyl laboratories) was used to measure the albumin production, Urea assay kit (BioAssay Systems) to measure the urea production of HLCs and TG quantification kit (BioVision) to measure the TG production from 24h conditioned medium and normalised to the total protein concentration (measured by Bradford assay; Thermo Scientific). To determine the lipid storing capability of the HLCs, the cells were stained by Oil red O staining and mounted with Vectashield (Vector Laboratories Inc., Burlingame, CA, USA) containing 40,6-diamidino-2-phenylindole (DAPI) for the nuclei staining and imaged with an Olympus IX51 phase-contrast microscope equipped with fluorescence optics and an Olympus DP30BW camera (Olympus Corporation, Hamburg, Germany).

Global Run-On sequencing (GRO-seq)

For GRO-seq, we collected nuclei of iPSCs from cell line UTA.11104.EURCAs, of M1-HLC from cell line UTA.11104.EURCAs and of M2-HLC from cell lines UTA.10100.EURCAs, UTA.11104.EURCAs, and UTA.11304.EURCCs. The nuclear run-on and library preparation was performed Cells were harvested by washing them with 1xPBS, detaching with Gentle Cell Dissociation reagent (STEMCELL™ Technologies, France), pelleting at 400xg, 5min at +4°C and finally resuspending in cold 1xPBS. The nuclei extraction was done as previously described [8]. For each replicate, 1-5 million cells were suspended to a final volume of 80-100 µl of freezing buffer.

The nuclear run-on and library preparation was performed the next day as described earlier [9]. The RNA was extracted using Trizol LS (Life Technologies, Carlsbad, CA), fragmented 13 mins in 70°C using RNA Fragmentation Reagents (Life Technologies) and purified by running through RNase-free P-30 column (Bio-Rad, Hercules, CA). The RNA was dephosphorylated with PNK for 2 hours (New England Biolabs, Ipswich, MA) followed by heat-inactivation. Dephosphorylation reactions were purified using 65 µl of blocked (5x volume of 0.25xSSPE, 1 mM EDTA, 37.5 mM NaCl, 0.05% Tween-20, 0.1% PVP and 0.1% ultrapure BSA for 1hour in RT) anti-BrdU bead slurry (SantaCruz Biotech, Santa Cruz, CA) suspended in 500 µl of binding buffer (0.25xsSSPE, 1 mM EDTA, 37.5 mM NaCl 0.05% Tween-20). After binding for 1 h in RT, the beads were washed twice with binding buffer, twice with low salt buffer (0.2xSSPE, 1mM EDTA, 0.05% Tween-20) and once with high salt buffer (0.2xSSPE, 1mM EDTA, 135 mM NaCl0.05% Tween-20) and twice with TE-buffer (1xTE, 0.05% Tween-20). The RNA was eluted three times by using 130 µl of elution buffer (50 mM Tris-HCl pH 7.5, 150 mM NaCl, 0.1% SDS, 1mM EDTA and 20 mM DTT) followed by ethanol precipitation overnight. All buffers were supplemented with SUPERase In (2 µl/10ml; Life Technologies). The libraries were amplified for 14 cycles and the final product of 190-350bp was extracted from on 10% Novex TBE gel (Life Technologies) and eluted from crushed gel slice twice using 100µl of elution buffer (TE+0.1% Tween+150 mM NaCl). The libraries were purified using ChIP DNA clean & Concentrator Kit (Zymo Research Corporation, Irvine, CA), quantified using the Qubit fluorometer and sequenced using Illumina HiSeq 2000 at EMBL Genecore, Heidelberg, Germany.

Processing of sequencing data and analysis of differentially regulated genes

The quality of reads was confirmed using FastQC tool (Andrews 2010; Available online at: http://www.bioinformatics.babraham.ac.uk/projects/fastqc) and poor quality reads were removed (minimum 97% of bp over phred quality cutoff 10) using the FastX toolkit (http://hannonlab.cshl.edu/fastx_toolkit/).

HOMER programs “analyzeRepeats.pl” and “getDiffExpression.pl” with the parameter “-batch” were used to get a list of differentially transcribed genes in the samples while treating them as biological replicates utilizing edgeR. Protein coding genes were regarded as differentially expressed when absolute log2 FC > 2 and adjusted p-value ≤ 0.05. The subset of differentially expressed transcript factors (TFs) was obtained by comparing list of differentially expressed genes to a list of known TFs. The list was downloaded from Ensembl database with biomart using the ontology term “DNA binding transcription factor activity” (GO:0003700) as a filter.

Comparison to publicly available transcriptome data

We utilised the normalised expression data of two publicly available microarray datasets (GSE14897 [1]; GSE61287 [10]), which were retrieved from GEO[11] using R-package GEOquery[12]. To yield expression estimate for a gene, the average intensity across the probe sets associated with that gene was calculated. For the dataset produced by Si-Tayeb *et al.* (GSE14897[1]) the log2Fold changes between HCLs and iPSCs were calculated using Limma[13]. Because the dataset produced by Takayama *et al.* (GSE61287 [10]) did not contain any replicates, the log2Fold change was calculated directly from the gene-level expression estimates between HCLs and iPSCs. In addition, a publicly available RNA-seq dataset (GSE10378 [14]) was included in the comparison, and the raw sequencing data was retrieved from NCBI′s Sequence Read Archive (SRA) database with the accession number SRP116261. The reads were aligned against hg19 reference genome using STAR[15] and the expression estimates were counted for RefSeq[16] gene annotation retrieved using UCSC table browser[17]. FeatureCounts[18] was used for counting the reads. The expression data was normalized and the log2FoldChanges for the fibroblasts and HCLs were counted using R-package DESeq2[19].

Quantification of pri-miRNA transcripts and identifying potential miRNA targets

We have previously annotated pri-miRNAs across 27 human cell types, including stem cells and liver hepatocellular carcinoma HepG2 cells [20]. We now utilized those coordinates to quantitate the pri-miRNA expression in the GRO-seq data and perform differential expression (log2FC > 1 or < -1, adj. p-value < 0.05) analysis on pri-miRNAs that had FPKM>0.5 in at least one sample group. miRNet tool [21] was used to perform visualization of miRNA-target networks and identify hub pri-miRNAs. Analysis was ran using defaults settings, but focusing analysis on liver tissue and only targets validated by CLIP studies. The contribution of each transcription start site (TSSi) to the overall transcriptional activity in a given locus was determined by subtracting the signal level at

the upstream element (TSSi+1), based on the FPKM values. The obtained values are referred to as differential TSS activity levels [20].

Identification of novel lncRNAs and quantification of lncRNA expression

LncRNA transcripts were detected for each sample using Homer´s 'findPeaks' command with default parameters. A consensus transcript set for the whole sample set was built by first collapsing all peaks that were within 200bp distance of each other to form a single peak using bedtools 'merge' command. Subsequently, peaks that were on the opposite strands and within 200 bp distance were clustered together using bedtools' 'cluster' command. The clustered peaks originating from opposite strands were merged together and classified as bidirectional (BI) transcripts, whereas the unclustered peaks were considered unidirectional (UNI) transcripts. For the BI transcripts, we estimated all putative TSSs by placing a TSS between the adjacent clustered peaks at the middle of the region defined by the ends of the adjacent clustered peaks that were most proximate to one another. To filter out known transcripts, we used bedtools' 'intersect' command to find transcripts that overlapped known RefSeq genes, lncRNA annotated in lncipedia and the pri-miRNA sites. For each transcript, we determined the closest protein-coding gene and the distance to that gene by using bedtools' 'closest' command. Based on the distance to the nearest gene, all transcripts located ≤ 10,000bp downstream of a coding gene were filtered out leaving only novel transcripts related to promoters and intergenic regions. The expression of each known and novel lncRNA was quantified and normalized with Homer´s 'analyze_repeats.pl' and the differentially expressed transcripts were detected with 'getDiffExpression.pl' using edgeR. lncRNAs were regarded differentially expressed when absolute log2FC > 2 and adjusted p-value ≤ 0.05, minimum read count was set at 20.

Annotation of known and novel non-coding transcripts to promoter- and enhancer-associated lncRNAs

Each lncRNA was assigned to one of two types: promoter- or enhancer-associated. To this end, available histone modification data was utilized [22]. The histone dataset comprised of two cell lines: H1-hESC and HepG2 for which the histone marks H3K4me1 and H3K4me3 had been quantified using ChIP-seq (UwHistone and BroadHistone data available via ftp://hgdownload.cse.ucsc.edu/goldenPath/hg19/encodeDCC/). For each TSS region comprised of

1000 bp up- and downstream from the TSS, the H3K4me1 vs H3K4me3 log2FC was quantified using Homer commands: “analyseRepeats.pl” and “getDiffExpression.pl” by treating the different cell lines as replicates. Each transcript was then assigned as promoter-associated if the log2FC > 0 and enhancer-related if log2FC < 0. If log2FC = 0, the type was determined by the distance and orientation to the closest coding gene. When the lncRNA was located in the upstream region of the closest coding gene, it was considered as promoter- associated if its distance from the coding gene was ≤ 3,000 bp and enhancer-associated otherwise. If lncRNA was located in the downstream region of the closest coding gene, it was considered promoter-associated if the distance to the closest gene was ≤ 10,000 bp and enhancer-associated otherwise. Bedtools "intersect" command was used to find overlaps between the lncRNAs and the coordinates of known super-enhancers (SEs) discovered in the HepG2 cells [23].

Clustering analyses and visualization of expression

To compare the expression profiles across the samples, a clustering analysis was conducted using the pheatmap-package in R. Prior to clustering, we filtered out the intragenic lncRNA and reduced redundancy of the transcript set by selecting the longest transcript out of the overlapping ones. This set of transcripts is referred to as the summarized transcript set. Furthermore, low expressed transcripts were filtered out based on either maximum FPKM or normalized read counts across the samples. Also, protein coding genes with expression less than one FPKM and lncRNAs with normalized read count <20 reads across all samples were filtered out.

Correlation analyses

The correlation analysis was conducted on the summarized transcript set described above. In addition, transcripts that were located > 10,000 bp from the closest gene were filtered out prior to the analysis. The correlation between PHHs and HLCs was calculated and the dot-plots created using the ggplot packages in R.

Gene ontology enrichment analysis for differentially expressed genes and lncRNA

The gene ontology (GO) enrichment analysis was performed using WebGestalt (http://www.webgestalt.org/) [24]. All the protein-coding genes were used as a background set. GO terms with FDR < 0.05 were considered statistically significantly enriched. In addition, the minimum and the maximum number of genes associated to an enriched GO term was 5 and 2000, respectively.

Motif enrichment

To study which TFs were enriched at differentially expressed lncRNA TSSs, motif enrichment was studied using the HOMER 4.7 'findMotifsGenome.pl' command with the default settings and the number of motifs within each TSS was counted using the 'annotatePeaks.pl' setting -nmotifs.

Supplementary references

1. Si-Tayeb, K. *et al.* Highly efficient generation of human hepatocyte-like cells from induced pluripotent stem cells. *Hepatology* **51,** 297–305 (2010).
2. Agarwal, S., Holton, K. L. & Lanza, R. Efficient Differentiation of Functional Hepatocytes from Human Embryonic Stem Cells. *Stem Cells* **26,** 1117–1127 (2008).
3. Song, Z. *et al.* Efficient generation of hepatocyte-like cells from human induced pluripotent stem cells. *Cell Res.* **19,** 1233–1242 (2009).
4. Cai, J. *et al.* Directed differentiation of human embryonic stem cells into functional hepatic cells. *Hepatology* **45,** 1229–39 (2007).
5. Hay, D. C. *et al.* Highly efficient differentiation of hESCs to functional hepatic endoderm requires ActivinA and Wnt3a signaling. *Proc. Natl. Acad. Sci. U. S. A.* **105,** 12301–6 (2008).
6. Teo, A. K. K., Valdez, I. A., Dirice, E. & Kulkarni, R. N. Comparable generation of activin-induced definitive endoderm via additive Wnt or BMP signaling in absence of serum. *Stem cell reports* **3,** 5–14 (2014).
7. Kiamehr, M. *et al.* Lipidomic profiling of patient-specific iPSC-derived hepatocyte-like cells. *Dis. Model. Mech.* **10,** (2017).
8. Core, L. J., Waterfall, J. J. & Lis, J. T. Nascent RNA sequencing reveals widespread pausing and divergent initiation at human promoters. *Science* **322,** 1845–8 (2008).
9. Kaikkonen, M. U. *et al.* Control of VEGF-A transcriptional programs by pausing and genomic compartmentalization. *Nucleic Acids Res.* **42,** 12570–84 (2014).
10. Takayama, K. *et al.* Prediction of interindividual differences in hepatic functions and drug sensitivity by using human iPS-derived hepatocytes. *Proc. Natl. Acad. Sci. U. S. A.* **111,** 16772–7 (2014).
11. Edgar, R., Domrachev, M. & Lash, A. E. Gene Expression Omnibus: NCBI gene expression and hybridization array data repository. *Nucleic Acids Res.* **30,** 207–10 (2002).
12. Davis, S. & Meltzer, P. S. GEOquery: a bridge between the Gene Expression Omnibus (GEO) and BioConductor. *Bioinformatics* **23,** 1846–1847 (2007).
13. Ritchie, M. E. *et al.* limma powers differential expression analyses for RNA-sequencing and microarray studies. *Nucleic Acids Res.* **43,** e47 (2015).

14. Gao, Y. *et al.* Distinct Gene Expression and Epigenetic Signatures in Hepatocyte-like Cells Produced by Different Strategies from the Same Donor. *Stem Cell Reports* **9,** 1813–1824 (2017).

15. Dobin, A. *et al.* STAR: ultrafast universal RNA-seq aligner. *Bioinformatics* **29,** 15–21 (2013).

16. O'Leary, N. A. *et al.* Reference sequence (RefSeq) database at NCBI: current status, taxonomic expansion, and functional annotation. *Nucleic Acids Res.* **44,** D733-45 (2016).

17. Karolchik, D. *et al.* The UCSC Table Browser data retrieval tool. *Nucleic Acids Res.* **32,** D493-6 (2004).

18. Liao, Y., Smyth, G. K. & Shi, W. featureCounts: an efficient general purpose program for assigning sequence reads to genomic features. *Bioinformatics* **30,** 923–930 (2014).

19. Love, M. I., Huber, W. & Anders, S. Moderated estimation of fold change and dispersion for RNA-seq data with DESeq2. *Genome Biol.* **15,** 550 (2014).

20. Bouvy-Liivrand, M. *et al.* Analysis of primary microRNA loci from nascent transcriptomes reveals regulatory domains governed by chromatin architecture. *Nucleic Acids Res.* **45,** 12054 (2017).

21. Fan, Y. *et al.* miRNet - dissecting miRNA-target interactions and functional associations through network-based visual analysis. *Nucleic Acids Res.* **44,** W135–W141 (2016).

22. Consortium, T. E. P. An integrated encyclopedia of DNA elements in the human genome. *Nature* **489,** 57–74 (2012).

23. Hnisz, D. *et al.* Super-Enhancers in the Control of Cell Identity and Disease. *Cell* **155,** 934–947 (2013).

24. Wang, J., Vasaikar, S., Shi, Z., Greer, M. & Zhang, B. WebGestalt 2017: a more comprehensive, powerful, flexible and interactive gene set enrichment analysis toolkit. *Nucleic Acids Res.* **45,** W130–W137 (2017).

Supplementary Figures

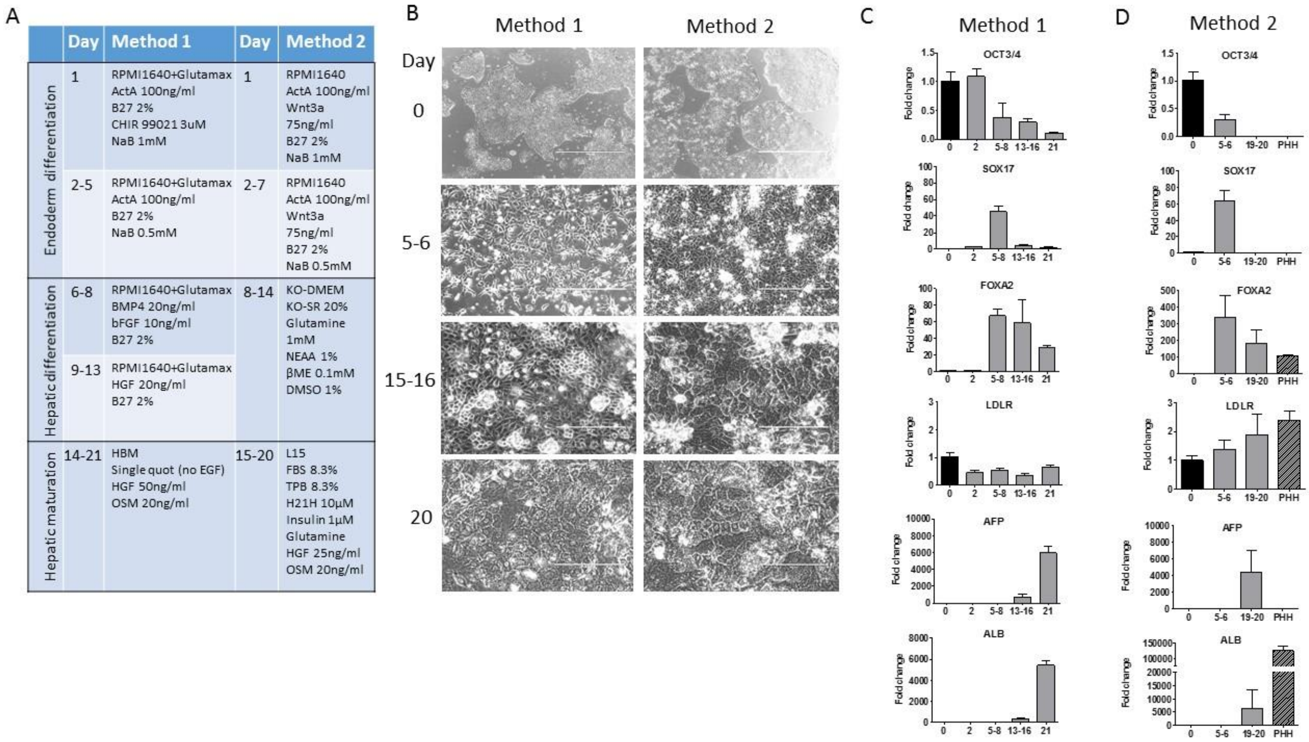

Supplementary Figure S1. Differentiation of induced pluripotent stem cells (iPSCs) to hepatocyte-like cells (HLCs) and temporal dynamics of specific genes during differentiation. Related to Figure 1. (A) Schematic representation of the two protocols (method 1, M1 and method 2, M2) used to differentiate the iPSCs to HLCs. (B) Images showing morphology of the cells at day 0 (iPSC colonies, 4x, scale bar 1000µm), day 5-6 (10x, 400µm), day 15-16 and day 20-21 (20x, 200µm) of the hepatocyte differentiation process starting from iPSCs. ActA, activin A; NaB, Sodium butyrate; bFGF, basic fibroblast growth factor; HBM, hepatocyte basal medium; HGF, hepatocyte growth factor; OSM, oncostatin M; NEAA, non-essential amino acids; βME, beta mercaptoethanol; DMSO, dimethyl sulfoxide; FBS, fetal bovine serum; TPB, tryptose phosphate broth; H21H, hydrocortisone 21-hemisuccinate. (C&D) Real-time qPCR showing downregulation of pluripotency marker Oct3/4, transient expression of endodermal marker genes SOX17 and FOXA2, upregulation of fetal and mature hepatocyte genes (AFP & ALB) and continuous expression of LDL receptor (LDLR) during the differentiation process from iPSCs to HLCs by method 1 (C) and method 2 (D). Gene expression was normalised to the housekeeping gene GAPDH, and expressed relative to iPSCs (=day 0). Each bar represents the mean ± SD from at least triplicate experiments. Some time-points were merged for the sake of clarity. iPSC, induced pluripotent stem cell; PHH, primary human hepatocyte; OCT, octamer-binding transcription factor; SOX17, Sry-related HMG box; CXCR4, C-X-C motif chemokine receptor 4; FoxA2, forkhead box protein A2; AFP, alpha fetoprotein; ALB, Albumin.

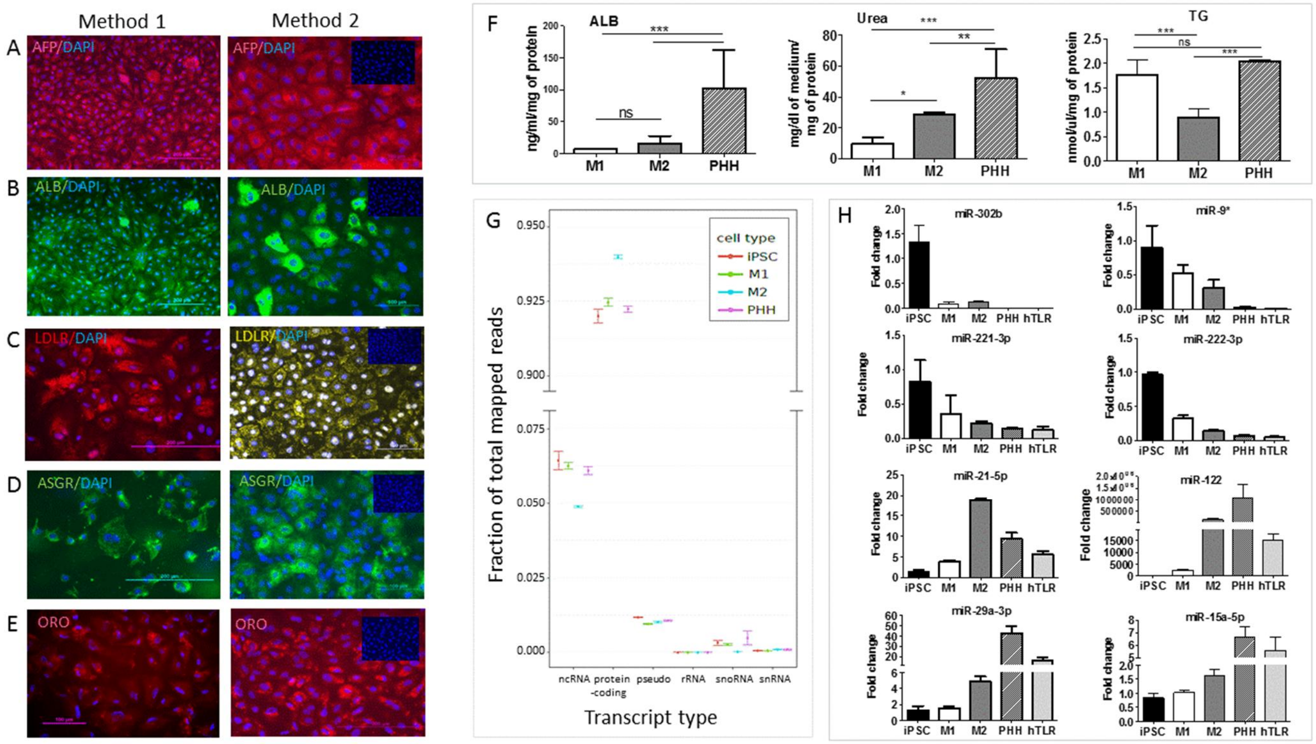

Supplementary Figure S2. Functional characterisation of iPSC-derived HLCs and expression of miRNAs in the iPSC, HLCs and PHHs. Related to Figure 2. Immunocytochemical staining of HLCs differentiated with method 1 and 2 showing the expression of alpha fetoprotein (AFP) (A), albumin (ALB) (B), LDL receptor (LDLR) (C) and asialoglycoprotein receptor (ASGR) (D). (E) Oil red O (ORO) staining was used to visualise lipid storing capability of the HLCs. Nuclei are stained with DAPI and Scale bars represent 100 or 200 µM. (F) Production of Albumin, Urea and triglyceride (TG) was measured at day 21 (M1) or day 19 (M2) from 24h conditioned medium and normalised to the total protein concentration (measured by Bradford assay; Thermo Scientific). (G) The fraction of mapped reads to different transcript types for iPSC, M1 (HLCs differentiated with method 1), M2 (HLCs differentiated with method 2) and PHH groups shown in red, green, blue and purple, respectively. Spheres represent the mean of mapped reads across replicates, and error bars illustrate the first and third quantile. (H) Expression of miR-302-b, miR-9*, miR-221-3p, miR-222-3p, miR-21-5p, miR-122, miR-29a-3p and miR-15a-5p genes in iPSC, M1 and M2-HLCs, PHHs and hTLR (iPSC stage expression level was used as the reference for all comparisons). PHH, primary human hepatocyte; hTLR, human total liver RNA.

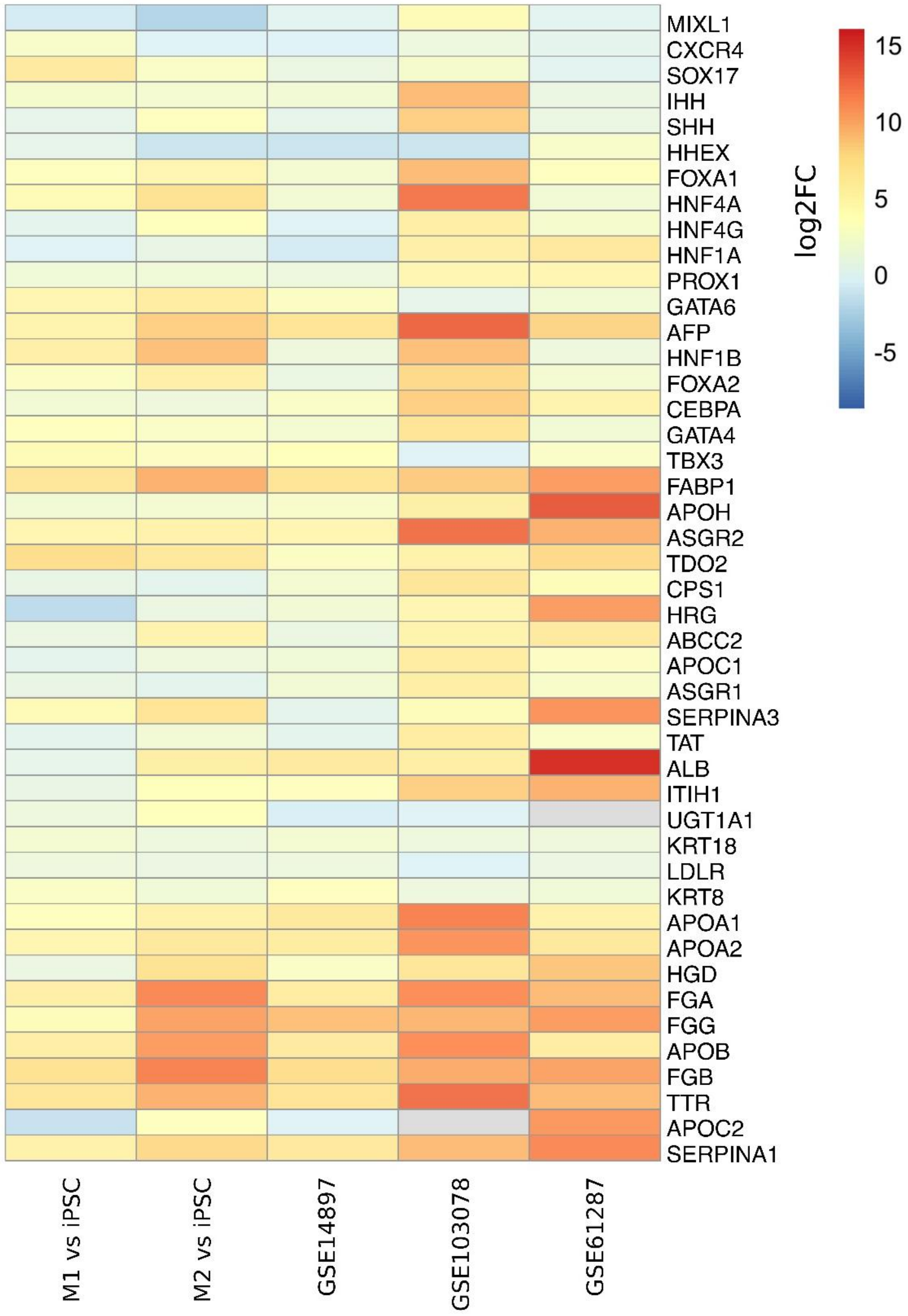

Supplementary Figure S3. Comparing the GRO-seq data of M1- and M2-HLCs to previously published transcriptome data. A heatmap representation of log2fold change in M1- and M2-HLCs vs. iPSCs in our GRO-seq study, in HLC vs. iPSCs in GSE14897 (corresponding to our M1), in HLC vs. UCFs in GSE103078 (corresponding to our M2) and in PHH-iPSC-HLCs vs. PHH-iPSCs in GSE61287. HLC, hepatocyte-like cell; iPSC, induced pluripotent stem cell; UCF, umbilical cord fibroblast; PHH-iPSC, iPSCs derived by reprogramming PHHs; PHH-iPSC-HLCs, HLCs differentiated from PHH-derived iPSCs.

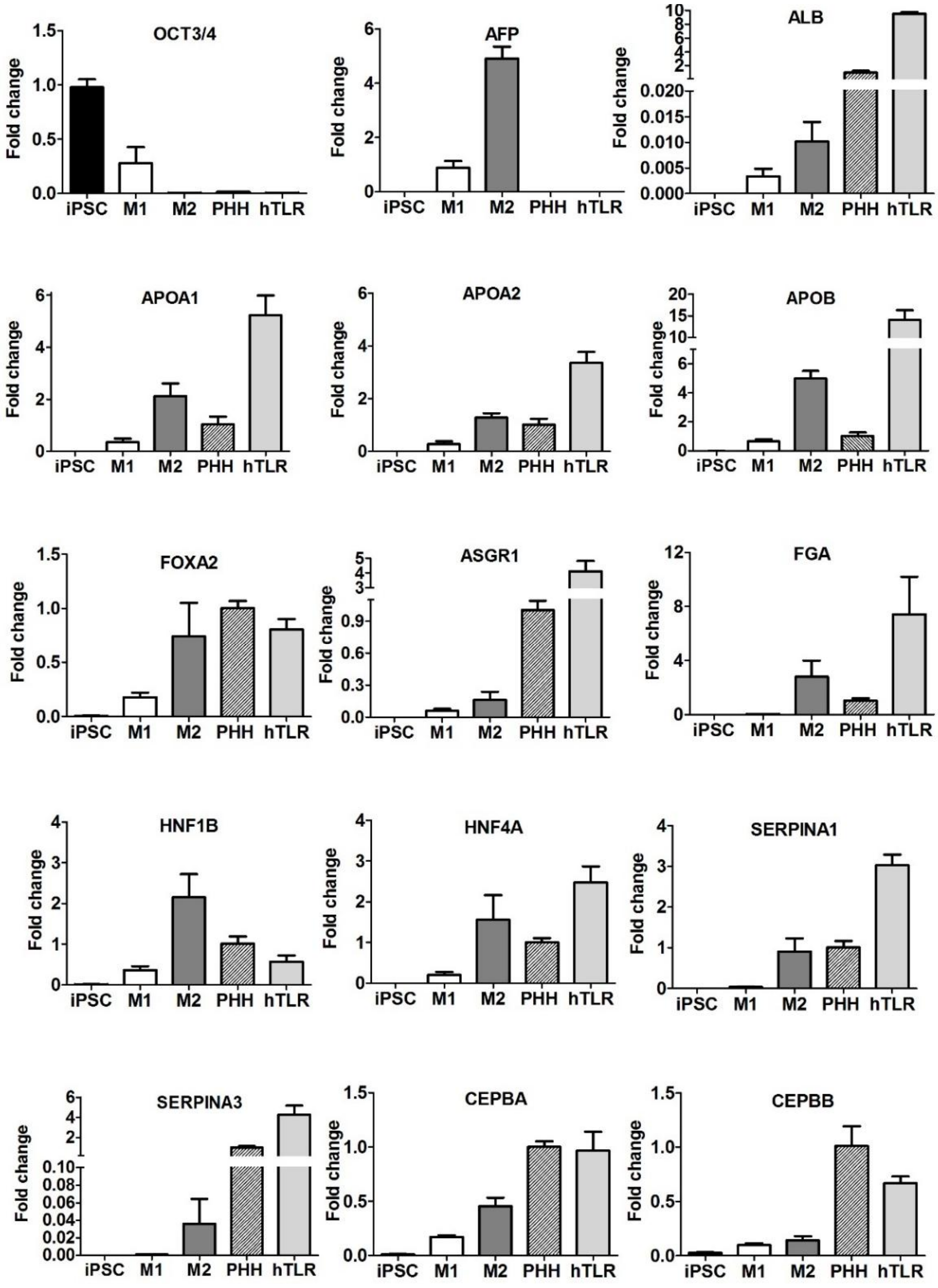

Supplementary Figure S4. Expression levels of important pluripotency and hepatic genes and transcription factors in iPSCs, M1- and M2-HLCs, PHHs and hTLR. Real-time quantitative PCR was used to measure gene expression in different groups. Expression levels were normalized to the housekeeping gene GAPDH, and are expressed relative to PHH. Each bar represents the mean ± SD from at least triplicate experiments. iPSC, induced pluripotent stem cell; M1, HLCs differentiated with method 1; M2, HLCs differentiated with method 2; HLC, hepatocyte-like cell; PHH, primary human hepatocyte; hTLR, human total liver RNA.

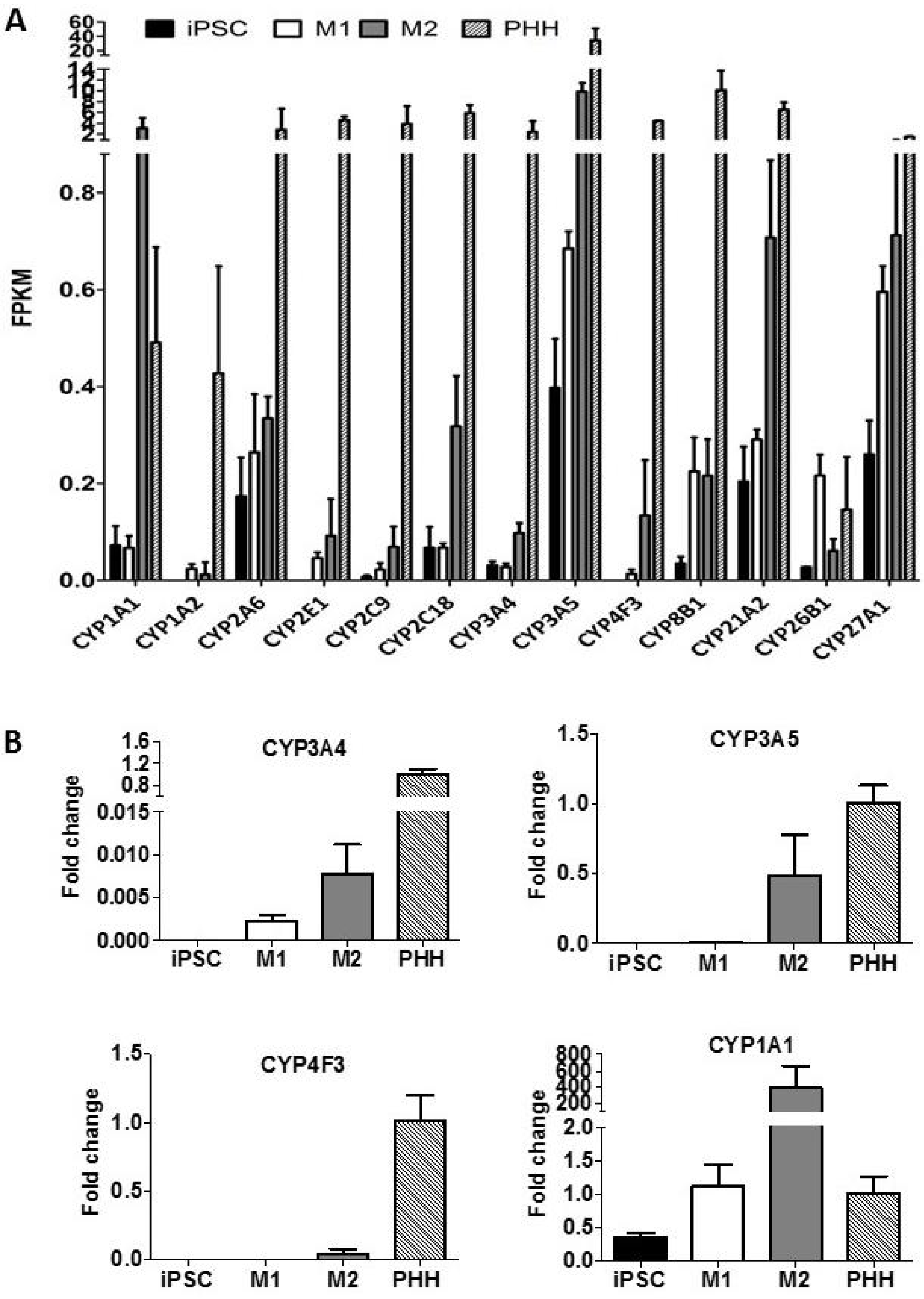

Supplementary Figure S5. The expression levels of ten Cytochrome 450 (CYP) enzymes in iPSCs, PHHs, M1- and M2-HLCs. Related to Figure 1. A) The CYP expression levels are presented as FPKM (= fragments per kilobase per million mapped reads = [# of mapped reads]/[length of transcript in kilo base]/[million mapped reads]). Bars represent mean ± SD. B) Validation of CYP4F3, CYP3A5, CYP3A4 and CYP1A1 expression levels by using TaqMan assays and real-time quantitative PCR. Bars represent mean ± SD. iPSC, induced pluripotent stem cell; M1-HLCs, HLCs differentiated with method 1; M2-HLCs, HLCs differentiated with method 2; PHH, primary human hepatocyte.

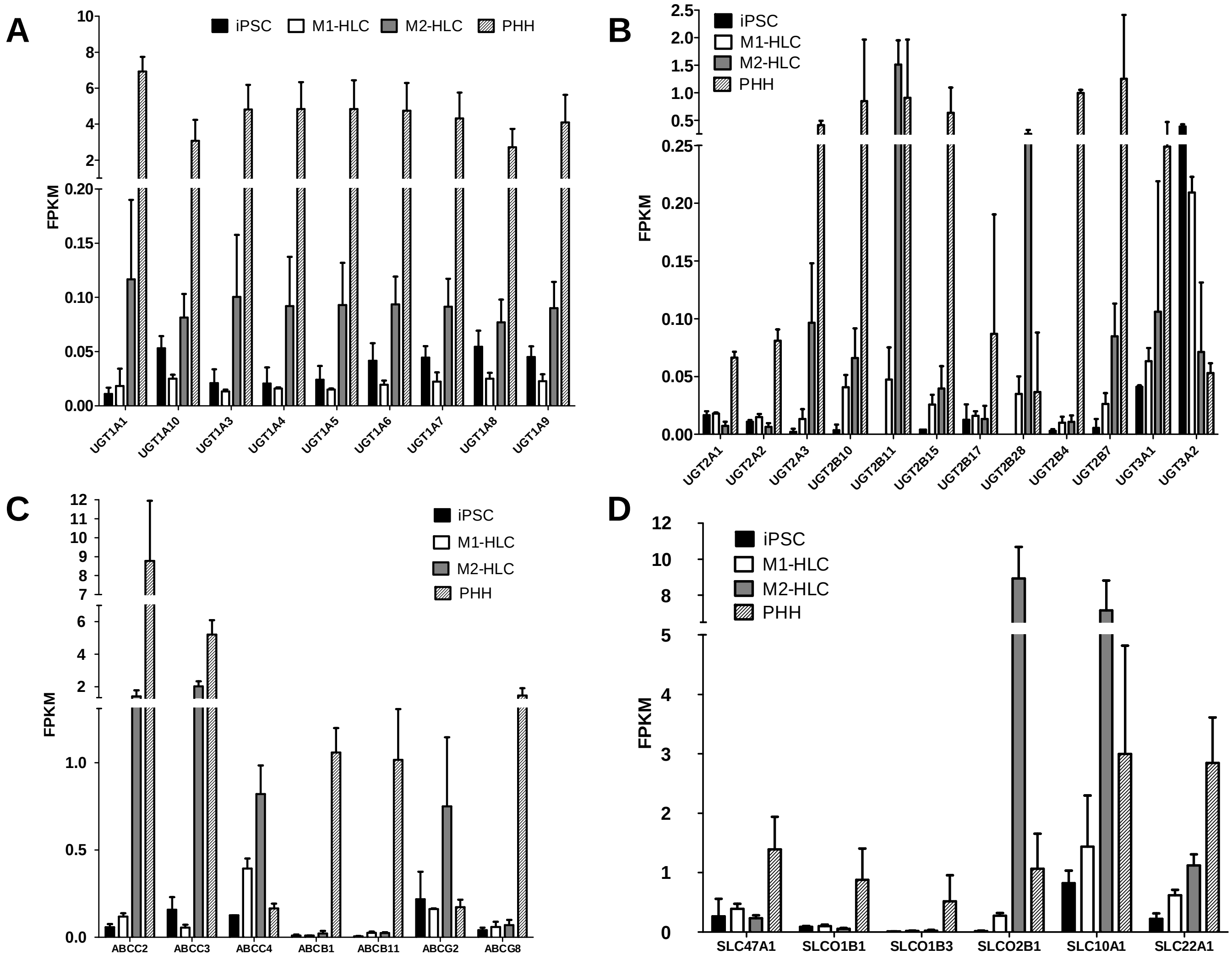

Supplementary Figure S6. Expression of 19 UDP-glucuronosyltransferases and three UDP-glycosyltransferases in iPSCs, PHHs, M1- and M2-HLCs. Related to Figure 1. A) The nine enzymes of the UGT1A family that were detected, were expressed at very constant levels within each sample group: the expression levels decreased in the order PHH>M2-HLCs>iPSC>M1-HLCs. B) The expression of UGT2A, UGT2B and UGT 3A enzymes. C&D) Highest expression of different transporter genes in the PHHs was detected for ABCC2 (encodes for MRP2 protein), ABCC3 (encodes for MRP3), SLC10A1 (encodes for NTCP) and SLC22A1 (encodes for OCT1), all of which had higher expression in M2- than M1-HLCs. The expression levels are presented as FPKM (= fragments per kilobase per million mapped reads = [# of mapped reads]/[length of transcript in kilo base]/[million mapped reads]). Bars represent mean ± SD. iPSC, induced pluripotent stem cell; M1, HLCs differentiated with method 1; M2, HLCs differentiated with method 2; PHH, primary human hepatocyte.
